# Influence of 8-weeks of supervised static stretching or resistance training of pectoral major muscles on maximal strength, muscle thickness and range of motion

**DOI:** 10.1007/s00421-023-05413-y

**Published:** 2024-01-19

**Authors:** Tim Wohlann, Konstantin Warneke, Vincent Kalder, David G. Behm, Tobias Schmidt, Stephan Schiemann

**Affiliations:** 1 Institute for Exercise, Sport and Health, Leuphana University, Lüneburg, Germany; 2 Institute of Sport Science, University of Oldenburg, Oldenburg, Germany; 3 Institute of Sport Science, Alpen-Adria-University Klagenfurt, Klagenfurt Am Wörthersee, Austria; 4 School of Human Kinetics and Recreation, Memorial University of Newfoundland, St. John’s, NL Canada; 5 Department of Sport Science, Medical School Hamburg, Hamburg, Germany

**Keywords:** Stretching, Flexibility, Resistance training, Tension

## Abstract

**Objectives:**

Current research suggests static stretch-induced maximal strength increases and muscle hypertrophy with potential to substitute resistance-training routines. However, most studies investigated the plantar flexors. This study explored the effects of a static stretching program on maximal strength, hypertrophy and flexibility of the pectoralis major and compared the effects with those of traditional resistance training.

**Methods:**

Eighty-one (81) active participants were allocated to either a static stretching, strength-training or control group. Pectoralis stretching was applied 15 min/day, 4 days per week for 8 weeks, while resistance training trained 3 days per week, 5 × 12 repetitions.

**Results:**

There was an increase in all parameters (strength: *p* < 0.001, *ƞ*^2^ = 0.313, muscle thickness: *p* < 0.001, *ƞ*^2^ = 0.157–0.264, flexibility: *p* < 0.001, *ƞ*^2^ = 0.316) and a time*group interaction (strength: *p* = 0.001, *ƞ*^2^ = 0.154, muscle thickness: *p* = 0.008–0.001, *ƞ*^2^ = 0.117–0.173, flexibility: *p* < 0.001, *ƞ*^2^ = 0.267). Post-hoc testing showed no difference between both intervention groups regarding maximal strength and muscle thickness (*p* = 0.905–0.983, *d* = 0.036–0.087), while flexibility increased in the stretching group (*p* = 0.001, *d* = 0.789).

**Conclusion:**

Stretching showed increases in maximal strength and hypertrophy, which were comparable with commonly used resistance training. Based on current literature, the influence of mechanical tension as the underlying mechanism is discussed. Furthermore, as equipment and comparatively long stretching durations are requested to induce meaningful strength increases in recreationally active participants, practical application seems limited to special circumstances.

## Introduction

Static stretching increases joint range of motion (ROM) (Konrad et al. 2023). While commonly associated with resistance training (Schoenfeld et al. 2017), recent studies in the literature demonstrated static stretching performed for several weeks to have the potential to induce increases in maximal strength (Arntz et al. 2023; Medeiros and Lima 2017) and muscle hypertrophy (Panidi et al. 2023). However, to induce relevant adaptations with stretch training, authors pointed out the need for high stretch intensities (Panidi et al. 2023), high volume, and long durations (Arntz et al. 2023; Panidi et al. 2023). For instance Panidi et al. (2023) showed higher stretch intensity to be more effective compared to lower intensities to induce muscle hypertrophy. Most recently, Warneke et al. (2023a, b, c) suggested static stretching as a potential alternative to common resistance-training methods, as the authors were not able to obtain significant differences in strength adaptations, muscle hypertrophy and flexibility when comparing 1 h of daily stretching with a commonly performed hypertrophy training routine (5 × 12 repetitions, three times per week). As resistance training can also improve ROM to a similar extent as static stretching (Alizadeh et al. 2023), the practical applicability and additional benefit of 1 h stretching per muscle group must considered critically (Schoenfeld et al. 2022). Furthermore, stretch training evidence is limited to studies mostly addressing lower extremity muscles (Warneke et al. 2023b).

However, the influence of static stretching on upper body maximal strength, such as with the pectoralis major and minor muscles is limited. To the best of our knowledge, only two studies explored the effects of pectoralis major stretching with three stretching exercises, each lasting 5 min on 3 days (Reiner et al. 2023) and 4 days (Warneke et al. 2023a) per week for 7 and 8 weeks, respectively. They found significant stretch-induced maximal strength and flexibility increases. However, no data on supporting morphological adaptations, such as muscle hypertrophy, contributing to maximal strength increases were collected. In resistance-training research, the influence of different load control parameters such as intensity is extensively investigated. Stretching intensity is commonly quantified subjectively by using an individual’s pain perception (2021; Panidi et al. 2023) which seems to be of limited validity (Lim and Park 2017).

Based on previous literature (Reiner et al. 2023; Warneke et al. 2023a), it was hypothesized that static stretching performed on the pectoral muscles (15 min, 4 days per week) can increase maximal strength, muscle thickness and flexibility. To check the practical relevance, the effects were compared with commonly performed resistance training. Furthermore, since strength training performed over the full ROM was reported to increase ROM (Alizadeh et al. 2023), both interventions are expected to induce significant shoulder ROM.

## Methods

### Experimental approach to the problem

To investigate the research question, physically active subjects were recruited and assigned to either a stretching group, strength training group or control group. The stretching group underwent a supervised 8 week—stretching training for pectoralis major muscle on 4 days per week for 15 min each session. Strength training group performed a commonly resistance training on 3 days per week for also 8 weeks, whereas no intervention was used in control group. Participants of all groups attended three laboratory sessions including an initial briefing, a pre- and post-test. The briefing session was also used for familiarization with the strength testing. In the pre- and post-tests, maximal isometric strength, muscle thickness, and shoulder ROM were measured.

### Subjects

Sample size estimation was performed using G-Power, based on previous research effect size of *f* = 0.25 (Warneke et al. 2023a). Considering *α* error to be 0.05 with a Power (1 − *β* err) = 0.8 for three groups and two measurements a total sample size of 42 was estimated. To counteract potential dropouts and increase the power, 81 recreationally active participants were recruited from university sports center and the university fitness center. Participants with injuries and surgery in the chest or shoulder during the last 6-month were excluded. Furthermore, to improve homogeneity within the sample, participants who reported performing daily stretching for the chest/shoulder were excluded from the study. All subjects were engaged in physical activity at least twice a week, participating in a wide range of sports, including such as fitness training, team sports, or strength-endurance training. All participants were instructed to maintain their regular training routine throughout their participation in the study. The characteristics are shown in Table 1. This study was performed in line with the principles of the Declaration of Helsinki. Approval was granted by the Oldenburg Ethics Committee 2022-064.

**Table 1:** Characteristics of participants (*n* = 81)

Group	*N* (male/female)	Age (year)	Height (cm)	Weight (kg)
SST	27 (17/10)	23.6 ± 2.5	178.0 ± 9.0	74.1 ± 14.0
STG	27 (18/9)	24.6 ± 4.2	178.4 ± 8.5	75.3 ± 12.9
CG	27 (17/10)	23.7 ± 2.8	179.1 ± 8.3	75.1 ± 12.1

*SST* static stretching training, *STG* Strength training, *CG* Control group

### Procedure

A standardized warm-up program consisting of 5 min of ergometer cycling (60 rpm) and 3 × 5 push-ups (or kneeing push-ups) had to be accomplished before testing.

### Maximal isometric strength tests

**Fig. 1:**
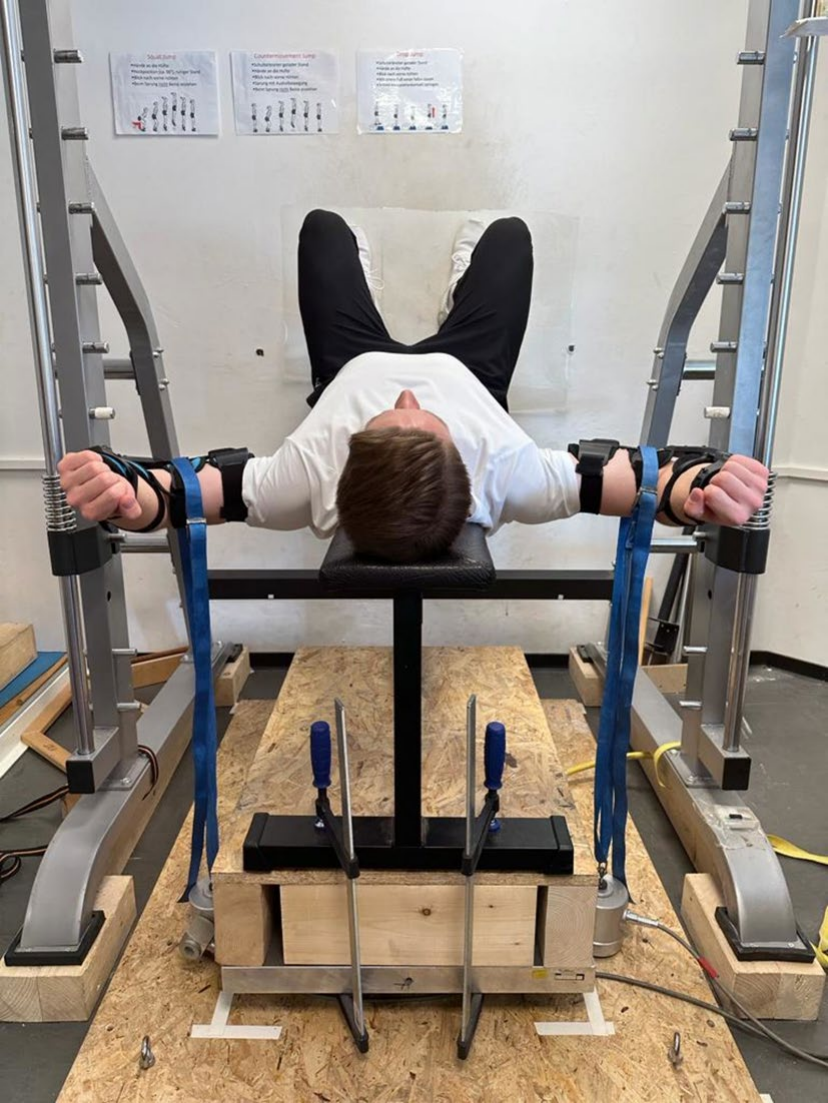
Measurement of maximal strength of pectoralis muscle

Isometric maximum strength was tested unilaterally for left and right pectoralis major muscle. Maximal strength values were summarized for further statistical calculations. The participants were positioned on a bench in the starting position of the butterfly exercise. The elbow joint was fixed to ensure the safety of the participants and a standardized testing procedure. A band was strapped over an orthosis and connected to a force transductor (Erichsen 56 Wuppertal 2, Type 19—02) (Fig. 1). Participants performed as many trials until the strength values dropped, with a minimum of three trials. A 120 s rest between trials was ensured to avoid fatigue.

### Measuring muscle thickness of pectoralis major

**Fig. 2:**
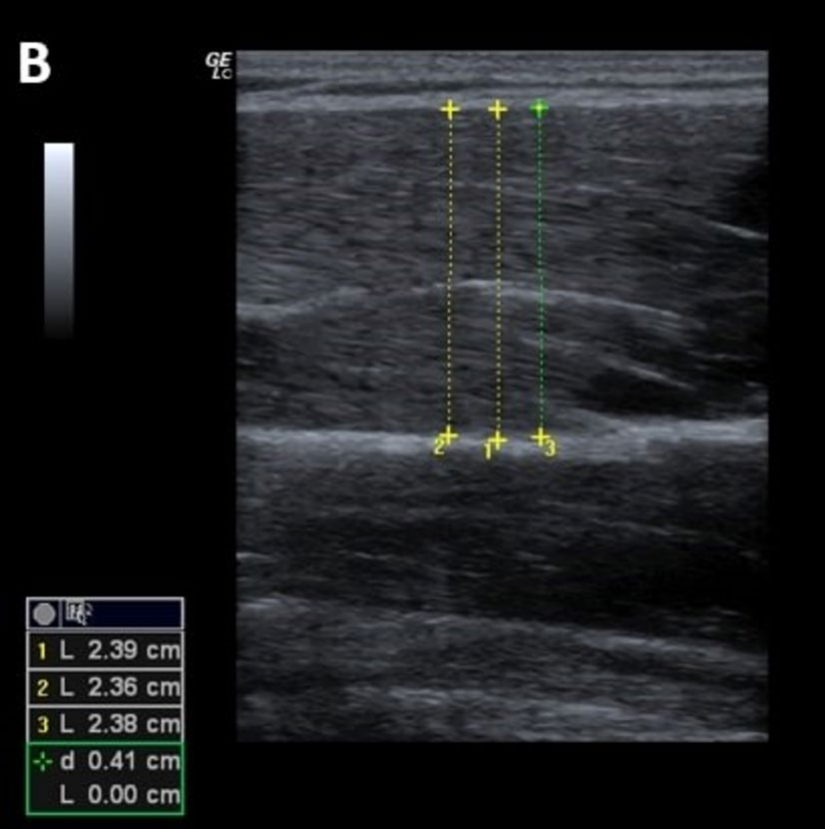
Sonography distance measurement of muscle thickness of pectoralis major muscle

Muscle thickness was examined using ultrasound imaging (LOGIQ C5 Premium device from GE medical Systems with a 5–14 MHz linear probe) of the pectoralis major. For this purpose, participants had to lie in a supine position on a medical bed, arms positioned in a relaxed position beside the body, with hands, shoulders and the head in a neutral position. The transducer was held above the axillary toward the acromion so that the pectoralis major was visible. Ultrasound was performed by a knowledgeable investigator with experience in ultrasound muscle thickness assessment. Two images of pectoralis major were acquired with three subsequent distance measurements centered in the image per test (Fig. 2). The average value of both individual muscle thickness images (each three distance measurements) was processed for further statistical calculation. Reliability of ultrasound measurement for the pectoralis major was reported to be high with ICC = 0.95 (Kotarsky et al. 2018), which was confirmed by reliability values calculated for this study (Table 2).

**Table 2:** Reliability for the pre-test values between best and second-best values

Parameter	ICC [95% CI]	CV in %
Maximal strength test—Butterfly	0.993	1.7–2.4
Muscle thickness—Sonography	0.966–0.961	1.1–2.0
Range of motion test—Shoulder pass through	0.995	1.3–4.3

*ICC* Intraclass correlation coefficient, *CV* Coefficient of variance

### Range of motion

**Fig. 3:**
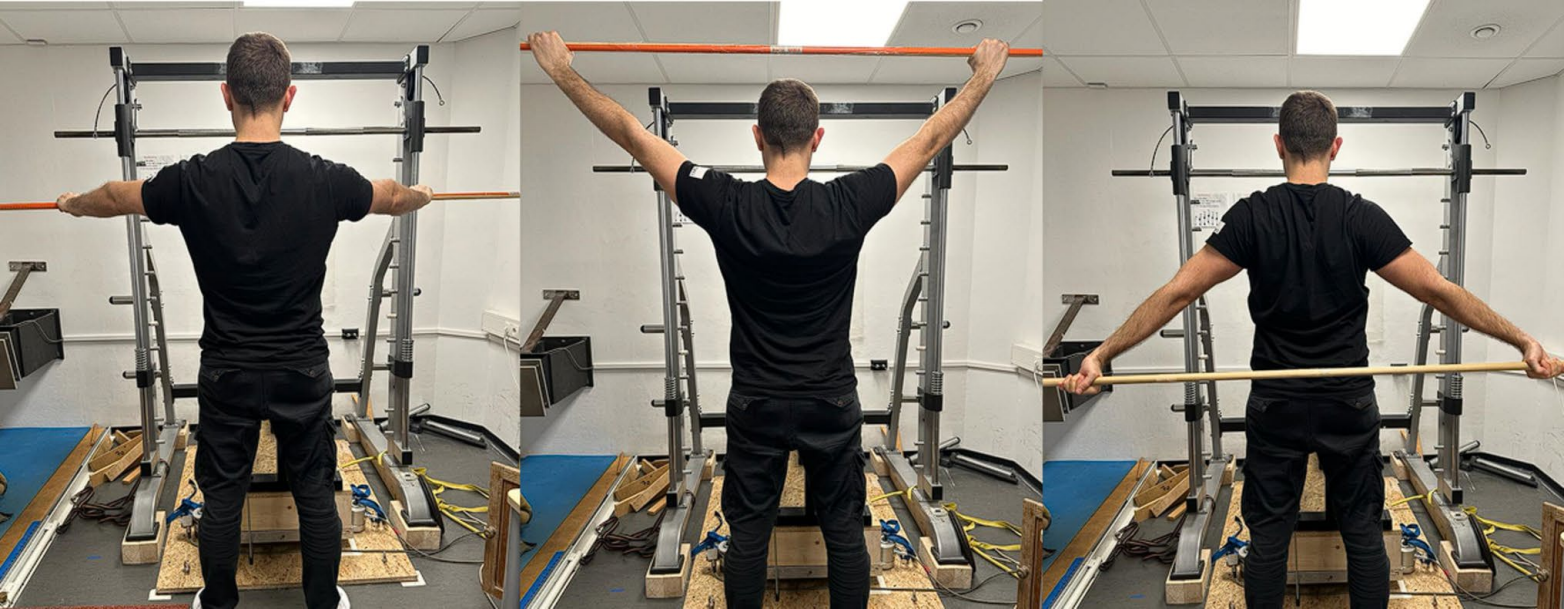
Measurement of the range of motion (ROM) test

For the shoulder ROM, the same test was used as in Warneke et al. (2023a). Participants held a bar in front of their body passed it backwards over their head and back again with arms extended. In the center of the rod were two markings in centimeters. The participants were instructed to position their hand at the number given to them by the instructor and recognizable on the inside of the hand. The trial was failed as soon as the elbows were flexed or the shoulder did any evasive movement during the movement (Fig. 3). The previously valid attempt was noted. Reliability of this procedure can be assumed to be reliable (ICC = 0.997–0.998) (Warneke et al. 2023a).

### Intervention

**Fig. 4:**
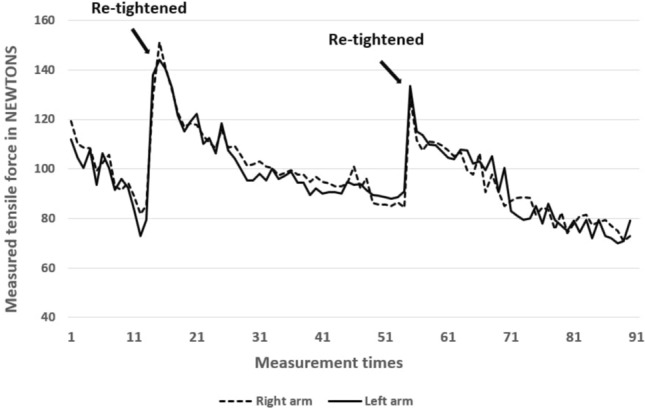
Measurement of 15 min of stretching with retightening. Stretching intensity was measured every 10 s

Participants in the stretching group performed supervised, continuous 15-min static stretching training 4 days per week over 8 weeks on a stretching board. Stretching volume was determined based on the protocol described by Warneke et al. (2023a). For the stretching, the participants were positioned on a bench with shoulders externally rotated and arms abducted at 90°, while the elbows were fixed at 90°. To avoid a hollow back, the legs were placed against a wall (Fig. 1). For the stretching, a ratchet strap was attached to the elbow joint and was connected to the force transducer that measured the applied tension every 10 s. The participants experienced a maximum tolerated stretching discomfort in the chest muscles. Since the measured mechanical tension decreased continuously over the period of 15 min, an automatic ratchet strap was used to retighten continuously to counteract relaxation induced mechanical tension loss applied to the muscle (Fig. 4).

To contrast high-volume stretch training to commonly performed resistance training, participants of the strength-training group performed standardized resistance training of the chest muscles 3 days per week for 8 weeks. Assuming the butterfly machine exercise to target the pectoralis major (Giorgio et al. 2009), the machine butterfly was performed (5 × 10–12 repetitions using the 10–12RM with 90 s interset rest). Training weight increased when participants reached 12 repetitions at every set.

### Statistical analyses

Statistical analysis was conducted utilizing SPSS 28 (IBM SPSS Statistics, version 28). Normal distribution was confirmed through the application of the Shapiro–Wilk test in pre-test values (*p* > 0.05). For reliability, intraclass correlations coefficient (ICC) and coefficient of variance (CV) were calculated for all groups (Table 2). Absence of pre-test group differences was tested by using a one-way analysis of variance (ANOVA). Two-way ANOVA with repeated measurements with the Scheffé post-hoc test was used to reveal significant differences within- and between groups. Pearson correlation *r* was calculated for maximal strength- and muscle thickness adaptations. Furthermore, effect sizes (eta square (*ƞ*^2^)) were categorized as small effect *ƞ*^2^ < 0.06, medium effect *ƞ*^2^ = 0.06–0.14 and large effect *ƞ*^2^ > 0.14. Differences in pre-to post-tests between the groups were reported using Cohen’s *d* with *d* < 0.5 = small effect, 0.5–0.8 = medium effect and > 0.8 = large effect (Cohen 1988). The critical level of significance was set at *p* = 0.05.

## Results

Reliability values were excellent for all measures (Table 2). There were no significant differences between pre-test values for all parameters.

### Isometric maximal strength

Both intervention groups showed high magnitude strength increases with a significant main effect (*p* < 0.001, *ƞ*^2^ = 0.31) and a Group*Time interaction (*p* = 0.001, *ƞ*^2^ = 0.15). Scheffé Post Hoc-Test revealed moderate magnitude, significant increases of the stretching (*p* = 0.01, *d* = 0.614) and strength group compared to control (*p* = 0.005, *d* = 0.664) respectively. No significant differences were obtained between the stretching and strength training group (*p* = 0.969, *d* = 0.049) (Table 3).

**Table 3:** Descriptive statistic and two-way ANOVA of all parameters

Group	Pre-test (mean ± SD)	Post-test (mean ± SD)	Change	Time effect	Time × group
Isometric maximal strength					
Stretching	461.3 ± 196.6 N	508.1 ± 207.1 N	+ 10.16%	*p* < 0.000 *F* = 35.495 *ƞ*^2^ = 0.313	*p* = 0.001 *F* = 7.110 *ƞ*^2^ = 0.154
Strength	493.3 ± 203.5 N	544.4 ± 188.6 N	+ 10.30%		
Control	475.5 ± 180.1 N	479.1 ± 179.4 N	+ 0.75%		
Muscle thickness pectoralis left					
Stretching	25.7 ± 7.3 mm	27.4 ± 7.4 mm	+ 6.46%	*p* < 0.001 *F* = 27.963 *ƞ*^2^ = 0.264	*p* = 0.001 *F* = 8.131 *ƞ*^2^ = 0.173
Strength	26.2 ± 5.4 mm	28.2 ± 5.8 mm	+ 7.25%		
Control	27.3 ± 6.6 mm	27.2 ± 6.4 mm	−0.31%		
Muscle thickness pectoralis right					
Stretching	25.7 ± 6.9 mm	27.2 ± 6.3 mm	+ 5.65%	*p* < 0.001 *F* = 14.561 *ƞ*^2^ = 0.157	*p* = 0.008 *F* = 5.185 *ƞ*^2^ = 0.117
Strength	26.3 ± 5.2 mm	27.7 ± 5.7 mm	+ 5.35%		
Control	27.5 ± 6.9 mm	27.3 ± 6.8 mm	−0.68%		
Range of motion					
Stretching	46.4 ± 11.8 cm	42.6 ± 11.2 cm	−8.86%	*p* < 0.001 *F* = 36.100 *ƞ*^2^ = 0.316	*p* < 0.001 *F* = 14.194 *ƞ*^2^ = 0.267
Strength	47.7 ± 7.8 cm	46.6 ± 8.3 cm	−2.38%		
Control	46.3 ± 9.9 cm	46.1 ± 10.2 cm	−0.48%		

### Muscle thickness

Large magnitude hypertrophy effects were obtained with a Time effect (*p* < 0.001, *ƞ*^2^ = 0.16–0.26) and a Group*Time interaction of *p* = 0.001–0.008, *ƞ*^2^ = 0.12–0.17). The Scheffé-Test showed moderate significant muscle thickness increases in the stretching (right: *p* = 0.018, *d* = 0.569; left: *p* = 0.007, *d* = 0.634), and strength group compared to the control (right: *p* = 0.029, *d* = 0.533; left: *p* = 0.002, *d* = 0.721), without a difference between the intervention groups (right: *p* = 0.983, *d* = 0.036; left: *p* = 0.905, *d* = 0.087) (Table 3).

### Range of motion

Large magnitude ROM increases were obtained with a Time effect (*p* < 0.001, *ƞ*^2^ = 0.32) and a large magnitude Group*Time interaction (*p* < 0.001, *ƞ*^2^ = 0.27). There were moderate magnitude significant ROM increases with stretching compared to strength training (*p* = 0.001, *d* = 0.789), while high magnitude increases were obtained comparing stretching to the control group (*p* < 0.001, *d* = 1.024). No difference was obtained between strength training and the control group (*p* = 0.492, *d* = 0.235) (Table 3).

### Relationship between muscle thickness increases and strength adaptations

Pearson correlation for pre-post changes in maximal strength versus muscle thickness showed correlations of *r* = 0.263; *p* = 0.018 (left side) and *r* = 0.203; *p* = 0.069 (right side).

## Discussion

This study compared the effects of 8-weeks of supervised static stretching with resistance training on strength capacity, muscle thickness and flexibility in the pectoralis major muscle. As hypothesized, static stretching and resistance training showed comparable results, demonstrating significant increases in maximal strength and muscle thickness, except for flexibility, which did not demonstrate a group difference. The results are in accordance with current evidence in human studies showing high-volume stretching can induce enhancements in strength capacity as well as muscle hypertrophy.

When explaining maximal strength increases, the literature provides different explanatory approaches such as functional, morphological and neuronal adaptations (Fleck and Kraemer 2004). In 2008, Goldspink and Harridge (2008) described the number of parallel sarcomeres (muscle cross-sectional area) to be a potential predictor for maximal strength increases. When seeking muscle hypertrophy and strength enhancements, resistance training is commonly used (Schoenfeld et al. 2017). Literature from 1970 to 2000 showed chronic stretching could induce morphological adaptations in chickens and quails (Warneke et al. 2022b). While Nunes et al. (2020) were not able to provide evidence for a transferability to humans including studies with a maximum of 5 min of stretching durations per session. Current human evidence has demonstrated stretch-mediated hypertrophy (Panidi et al. 2023) and increased strength capacity (Arntz et al. 2023) by using high stretching volumes and intensities. However, studies showing stretch-mediated hypertrophy were performed primarily in the lower extremities (Mizuno 2019; Panidi et al. 2021; Simpson et al. 2017; Warneke et al. 2022a, c, 2023c). Warneke et al. (2023c) and Reiner et al. (2023) were the only studies that showed significant strength increases in the upper body, but no data regarding hypertrophy were collected. Furthermore, there are no studies that have directly compared stretch and resistance training effects on muscle strength and hypertrophy in the human pectoralis muscle. Consequently, this study is the first that measured muscle hypertrophy in the upper extremities using stretching training with comparisons to resistance training.

Warneke et al. (2023c) suggested shared underlying physiological mechanism between stretching and resistance training by pointing out the relevance of high stretching tension as important to induce stretch-mediated hypertrophy. Muscle hypertrophy could be explained by translating mechanical tension into chemical signals that, in turn, stimulate anabolic processes such as satellite cells activation to generate new muscle tissue Tatsumi (2010). Accordingly, the role of mechanotransduction describing the translation of mechanical tension in biochemical signalling causing an anabolic response via the PI3K/AKT/mTOR signalling pathway was proposed. Furthermore, Apostolopoulos et al. (2015) described stretching intensity to be of crucial importance to induce structural muscle changes, hypothesizing stretched-mediated inflammatory processes. Indeed, reaching high degrees of stretching intensity could provide a sufficient stimulus to unfold titin filaments which can be hypothesized to be involved in the muscle hypertrophic response (Freundt and Linke 2019; Fukuda et al. 2008; van der Pijl et al. 2018). Some of these mechanisms were frequently suggested to be involved in muscle hypertrophy after resistance training as well (Lamas et al. 2010; Schoenfeld et al. 2022; Vissing et al. 2013; Wackerhage et al. 2019). Assuming mechanical tension to be of crucial importance, stretching intensity could be hypothesized to impact morphological adaptations (Panidi et al. 2023).

However, in most studies, stretching intensity is regulated by using individual pain scales such as a visual analogue scale and numeric pain scales (Nakamura et al. 2021; Warneke et al. 2022a; Wohlann et al. 2023). Lim and Park (2017) pointed out no correlation between measured passive tension and the subjective pain scale. Subjectively perceived stretching pain is influenced by various factors such as different sensory thresholds for pain, negative feelings, or physical conditions (Lim and Park 2017), leading to concerns regarding the objectivity of using subjective pain to manage intensity. To address concerns regarding intensity determination via subjective pain, a supervised static stretching program with a stretching device was performed. To ensure constantly high intensity stretch, in this study, mechanical tension was continuously re-adjusted and determined by using load cells. As shown in Fig. 4, the measured tensile force on the muscle continuously decreased, which made re-adjusting of the stretching intensity (ROM excursion) necessary to ensure high mechanical tension. Regarding the stimulus for maximal strength and hypertrophy, it can be speculated as to whether there is a subordinate role provided by either a single long-lasting mechanical tension (stretching) or recurring short mechanical tension (resistance training).

Apart from mechanical tension and morphological parameters, neural adaptations cannot be ruled out to be responsible for strength increases. In the literature, contralateral increases in maximal strength can be found after unilateral stretching training, indicating a neural influence (Nelson et al. 2012; Panidi et al. 2021; Warneke et al. 2022a). However, maximal strength in this study was tested bilaterally and no neuromuscular parameters were tested. Therefore, the discussion about neuromuscular adaptations contributing to stretch-mediated strength increases remain speculative. However, it is well investigated that training in general can lead to learning effects and influence maximal strength (Gabriel et al. 2006), especially in the early weeks of training (Del Vecchio et al. 2019).

The relevance of considering maximal strength increases as a multifactorial model is supported by obtained correlations for changes in maximal strength related to muscle hypertrophy with *r* = 0.2–0.26, *p* = 0.018–0.07. Even though significant, a correlation of 0.26 would explain about 6% of variance (Cohen 1988), hypothesizing a causal relationship. The limited practical/clinical relevance is underlined by the non-significant correlation of the right side, showing that the small correlation was not confirmed. Therefore, results are in line with Warneke et al. (2022a) providing no meaningful correlations between maximal strength- and muscle mass increases with *r* = 0.02, *p* = 0.9.

In the literature, there are many theories trying to explain an increase in flexibility or ROM after stretching. Some authors proposed the improved ROM by a reduction in pain perception (leading to increased stretch tolerance) (Freitas et al. 2018; Magnusson 1998), while others speculate about a change in muscle–tendon structure (Kruse et al. 2021). A recent systematic review with meta-analysis described stretching to reduce muscle stiffness in the long term (Takeuchi et al. 2023), while evidence for increases in serial sarcomere number in humans is still lacking (Zöllner et al. 2012).

### Practical applications

Regardless of the effects, practical applications of stretching are limited by some factors. Static stretch training via a stretching device like in the present study, made a second person necessary to assist the training program and to adjust the stretching device. Furthermore, regular resistance training can provide additional health benefits, such as the prevention of sarcopenia and osteoporosis (Holubiac et al. 2022; Hong and Kim 2018) and the improvement of cardiovascular health (Liu et al. 2019; Schjerve et al. 2008). While stretching seems to beneficially induce cardiovascular benefits (Thomas et al. 2021) stretching effects on bone density and sarcopenia were not explored in previous research. According to Schoenfeld et al. (2022), the practical application of using stretching to enhance muscle strength and cross-sectional area seems limited, since resistance training can be assumed to be more time efficient. However, Behm et al. (2023) described stretching as a potential alternative, if the resistance training hesitant is not willing to invest the effort in exercise sessions performed in the gym. Stretching might be applicable as a home-based training program (Warneke et al. 2023a). There might be situations without the possibility to perform more effective resistance training. In the COVID-19 Lockdown, 10 min of daily stretching for the calf muscle prevented performance losses (Warneke et al. 2022e). Furthermore, in situations such as post-surgery rehabilitation phases, stretching could also be a valuable supplementation of common therapy programs, if performed additionally.

### Limitations

The stretch-induced increases seem comparably high. The missing significant difference between the stretching and resistance training group regarding hypertrophy and strength increases might be attributable to an unknown training stimulus induced by the 15 min of continuous stretching or the low performance level of the included sample. In contrast, it can be assumed that most participants are accustomed to some kind of dynamic resistance training. Comparing effects of an unknown training stimulus to a familiar stimulus makes a final statement regarding the practical applicability difficult. Nevertheless, since resistance training can be considered more efficient (relationship between invested time and outcome), using long duration stretching seems exclusively applicable if no common training routine is possible. Furthermore, using load cells to quantify stretching intensity was not validated previously. Furthermore, not all participants were willing to join the stretching group, which prevented complete randomization. For participants who were indifferent to the group assignment, random allocation to one of the three groups was carried out. However, efforts were made to ensure an equal sex distribution, as well as training status. Furthermore, sonography for measuring hypertrophy should be interpreted critically (Warneke et al. 2022d), especially if using just one measurement point. It is recommended to apply more than one spot for measuring muscle thickness via sonography to increase validity (Nunes et al. 2023).

## Conclusion

In conclusion, this study indicated that 8-weeks of supervised static stretching (15 min, 4 days per week) performed for the pectoralis muscle induced comparable strength increases, muscle hypertrophy and ROM improvements compared to a commonly performed resistance training. Further research is required to clarify the underlying mechanisms as both, neural and structural adaptations may be responsible. The practical applicability is limited by the availability of stretching devices, spent time for stretching and considerable side effects.

## Data Availability

Original data can be provided upon reasonable request. The authors report that there are no competing interests to declare. The researchers have no financial interests.

## Abbreviations

ANOVA: Analysis of variance
CV: Coefficient of variance
ICC: Intraclass correlation coefficient
ROM: Range of motion
